# Pharmaco-EEG-Based Classification of Psychotropic Activity of a Novel Chromone-Containing Allylmorpholine in Rats

**DOI:** 10.34172/apb.025.45140

**Published:** 2025-10-11

**Authors:** Yuriy I. Sysoev, Nikita S. Kurmazov, Darya D. Shitc, Maria M. Puchik, Elizabeth V. Fedorova, Nikita V. Petrov, Nikita M. Chernov, Sergey A. Chervonetskiy, Sergey V. Okovityi

**Affiliations:** ^1^Saint Petersburg State Chemical and Pharmaceutical University, Saint Petersburg, Russian Federation; ^2^Pavlov Institute of Physiology, Russian Academy of Sciences (RAS), Saint Petersburg, Russian Federation; ^3^N.P. Bechtereva Institute of the Human Brain, Saint Petersburg, Russian Federation; ^4^Institute of Translational Biomedicine, Saint Petersburg State University, Saint Petersburg, Russian Federation

**Keywords:** Pharmaco-EEG, ECoG, Rats, Naïve Bayes classifier, Allylmorpholine derivatives, Chromone derivatives, Antipsychotic activity

## Abstract

**Purpose:**

Chromone-containing allylmorpholines (CCAMs) are a promising class of compounds that have been shown to have a dose-dependent inhibition effect on locomotion in zebrafish (Danio rerio). However, experiments using behavioural tests on mice have not yet allowed us to fully understand the specificity of their action. In this study, we conducted a pharmacoencephalographic evaluation of the psychotropic effects of CCAM 33a on rats using a Naïve Bayes classifier (NBC) combined with the principal component analysis (PCA).

**Methods:**

The ECoG experiments were conducted on white outbred rats. The training set, which was used as a reference for determining the pharmacological effects of each dose of the compound under study, included matrices of effects from 9 agents with different mechanisms of action. Amplitude-spectral analysis using PCA resulted in 6 new principal components that accounted for 83.57% of the variance. Classification of the effects of compound 33a was performed using NBC. To validate the classification results, additional experiments were conducted including the 5-hydroxytryptophan (5-HTP)-induced head twitch test and apomorphine-induced climbing in mice, as well as the ‘presynaptic’ low-dose apomorphine-induced yawning test in rats.

**Results:**

CCAM 33a at doses of 100 and 300 mg/kg shows similar effects to hydroxyzine and sulpiride. In mouse experiments, CCAM 33a reduced the number of head twitches induced by 5-HTP administration at a dose of 20 mg/kg, and inhibited apomorphine-induced climbing at a dose of 300 mg/kg. In rats, the substance at a dose of 100 mg/kg reduced the number of yawns caused by apomorphine administration at a dose of 0.032 mg/kg.

**Conclusion:**

The data obtained confirm the effectiveness of the combined use of NBC and PCA for classification tasks. The effects of the different doses of compound 33a on ECoG, as well as the abolition of the effects of apomorphine and 5-HTP in mice and rats, suggest a dopamine- and 5-HT2-blocking action of the molecule under study.

## Introduction

 The development of relevant methods for classifying the effects of psychotropic drugs and *in vivo* models is an important task in biomedicine. This is due to the fact that different groups of drugs can have similar physiological effects on experimental animals when using behavioural testing methods. For example, if a test drug reduces locomotor activity in animals, and it is difficult to determine its exact pharmacological action, this effect could be associated with anxiolytic, antipsychotic, sedative or even neurotoxic actions.

 Pharmaco-electroencephalography (pharmaco-EEG) is a field of pharmacology and electrophysiology that examines the effects of medications on the central nervous system. It does this by analysing changes in the parameters of bioelectrical activity of the brain (usually the cortex) in response to the administration of psychoactive substances. Pharmaco-EEG is one of the most promising methods for screening of psychotropic drugs due to the advent of selective receptor ligands of neurotransmitter systems and new methods for data dimensionality reduction, classification and prediction^1^. With the availability of a library of EEG effects of known drugs, machine learning algorithms can be used to identify the drug class^2-4^ and potentially determine the spectrum of its effects on specific neurotransmitter systems. These algorithms can also help to establish molecular targets, down to the subtype of a particular receptor. To process and interpret large amounts of pharmaco-EEG data, mathematical algorithms to reduce the dimensionality of the data (e.g. PCA — principal component analysis), and machine learning algorithms, for example, Naïve Bayes classifier (NBC), are used. NBC is a simple probabilistic classifier that considers each feature of classified data independently of other features. It has been widely used in medical practice for predicting patient resistance to pharmacotherapy,^5^ diagnosing diabetes mellitus^6^ or assessing the risk of drug-induced liver damage.^7^ Every year this algorithm is increasingly used for classification and prediction purposes and in other areas of biomedicine as well. The effectiveness of NBC has been demonstrated in classifying the effects of antipsychotic drugs based on their impact on EEG parameters in rats.^8,9^ However, further validation is needed to confirm its use as a tool for screening new understudied molecules with potential pharmacological activity.

 Chromone-containing allylmorpholines (CCAMs) are a class of compounds that have been shown to have the ability to inhibit acetyl- and butyrylcholinesterase and antagonism towards N-methyl-D-aspartate receptors in *in vitro *experiments.^10^ In the present study, (E)-4-[3-(6-chloro-4-oxo-4H-chromen-3-yl)-4-cyclohexylallyl]morpholin-4-ium chloride (33a) was selected as the most promising molecule among the compounds of this group. At low doses, this compound exhibited anxiolytic effects in *Danio rerio* in the Novel tank and Light/Dark box tests.^11^ In BALB/C mice, 33a had a locomotor-inhibitory effect at a dose of 50 mg/kg, but no specific action was observed at lower doses, e.g., in the Elevated Plus Maze or Tail Suspension tests.^12^ Therefore, a previously used approach^8,9^ was employed to determine the specificity of the action of 33a. The aim of this study is the pharmaco-EEG evaluation of the psychotropic activity of (E)-4-[3-(6-chloro-4-oxo-4H-chromen-3-yl)-4-cyclohexylallyl]morpholin-4-ium chloride (33a) in rats with subsequent validation of the method using traditional behavioural tests.

## Materials and methods

###  Animals

 Experiments using laboratory animals were conducted in accordance with the requirements of Directive 2010/63/EU of the European Parliament and the Council of September 22, 2010, as well as the principles of the Basel Declaration and the regulations of the Council of the Eurasian Economic Union, dated November 14, 2023, No. 33 “On Guidelines for handling laboratory (experimental) animals when conducting preclinical (non-clinical) studies”. The protocol for the experiment was approved by the Bioethics Commission of Saint Petersburg State Chemical and Pharmaceutical University of the Ministry of Health of the Russian Federation (protocol-application R-PEEG2-SA-2022 dated February 15, 2022). Every effort was made to reduce the number of animals used in the study and minimize their suffering.

 The study was performed on male Wistar rats aged 3 months and weighing 250–300 g (*n* = 112 — for the pharmaco-EEG study; *n* = 60 — for apomorphine-induced yawning test). Apomorphine-induced climbing test and 5-HTP-induced head-twitch test were performed on 60 outbred white male mice, aged 3 months and weighing 20–22 g. Animals were obtained from the “Kurchatov Institute” — Rappolovo Laboratory Animal Nursery (Leningrad Region, Russia). Rats were kept five per cage, and mice were housed ten per cage at a room temperature of 20–22 °C and with a 12-hour light/12-hour dark cycle. All animals were fed standard chow (dry, full-fed, granular, and extruded compound feed recipe PK120 from OOO Laboratorkorm, Russia) and had *ad libitum* access to food and water. Prior to the experiments, all animals were quarantined for 14 days.

###  ECoG electrodes manufacturing, implantation and post-operative animal care

 The stages of nichrome ECoG electrodes manufacturing, implantation, and post-operative animal care have been described in detail in a previously published paper.^13^ Tiletamine/zolazepam 100 (Zoletil, Virbac, France; 30 mg/kg, intramuscularly) was used to anesthetize the animals. Electrodes FP1 and FP2 were placed in the primary motor cortex area (AP = 0.0; ML = 2.5; DV = 1.0), while C3 and C4 were positioned over the primary somatosensory cortex near the hippocampus (AP = –4.0, ML = 2.5; DV = 1.0). Electrodes O1 and O2 were implanted in the secondary visual cortex region (AP = –7.0; ML = 2.5; DV = 1.0), with the reference electrode positioned in the nasal bone and the ground electrode implanted subcutaneously in the neck area.

 After the surgery, the rats were kept in individual cages and had *ad libitum* access to food and water throughout the study period. Their condition was assessed immediately after termination of anaesthesia and then daily in the morning and evening. If necessary, their sutures were treated with iodine solution. Bicillin-3 (Sintez, Russia; 5000 U/kg, subcutaneous injection) was given immediately after surgery to prevent infection, and ketoprofen (Velpharm, Russia; 2.5 mg/kg, subcutaneous injection once a day for 3 days) was administered to reduce postoperative pain. To prevent dehydration, normal saline (OOO Grotex, Russia; 5 mL subcutaneous injection once a day) was given to the rats during the first three days after surgery.

###  ECoG recording

 ECoG recordings in animals were performed at least one week after implantation using an 8-channel Neuron-Spectrum-1 encephalograph (OOO Neurosoft, Russia), with a frequency range of 0.5–35 Hz and a sampling rate of 500 Hz. Simultaneously, video recordings of the animal’s behaviour in its home cage under artificial lighting were made. The recording lasted for 1 hour and included 30 minutes of baseline activity (prior to drug or saline injection) and 30 minutes after injection. For further analysis, we took two 60-second recordings: one immediately before the injection and one 20 minutes after. Based on previous studies^8,9^ and our own experience with behavioural tests, most drugs that affect the central nervous system when administered intraperitoneally produce a noticeable effect within 20 minutes. However, this is not the case for drugs with a substrate mechanism of action, such as nootropics, or prodrugs. These were not used in our study. The selected samples contained ECoG recordings from rats in a quiet awake state. Motor activity, such as locomotion, rearing, grooming, or scratching, can significantly complicate the detection and differentiation of drug effects on brain bioelectric activity.^14^ The classification and prediction approach described in this paper, as well as in previous publications,^8,9^ does not limit the duration of ECoG samples. However, due to the high background activity in some rats, we chose a 60-second recording duration to ensure that the signal was clean from motor artefacts.

###  Drugs

 For ECoG recordings all the drugs used were administered intraperitoneally. If necessary, they were pre-dissolved in water for injections to the required concentration. Substance 33a, synthesised according to a previously published method^10^ by the Organic Synthesis Department of the Saint Petersburg State Chemical and Pharmaceutical University, was administered at doses of 50, 100, and 300 mg/kg. The training set, which was used as a reference to determine the pharmacological effects of each dose of the investigated substance, included the following drugs: NMDA-antagonist dizocilpine (Targetmol Chemicals Inc., USA), D2/D3-antagonists haloperidol (OOO Velpharm, Russia) and sulpiride (AO Organica, Russia), M-cholinergic receptor blocker tropicamide (Tocris Bioscience, UK), H1/5HT2A-receptor blocker hydroxyzine (Sigma-Aldrich Chemie GmbH, Germany), acetylcholinesterase inhibitor galantamine (Sopharma AD, Bulgaria), alpha-2 adrenomimetics dexmedetomidine (Orion Corporation, Finland), GABA-mimetics aminophenylbutyric acid (Tocris Bioscience, UK) and bromdihydrochlorphenylbenzodiazepine (phenazepam) (AO Novosibchimpharm, Russia) (Table 1). As a control, 0.5 mL of normal saline (OOO Grotex, Russia) was administered. The validation set included promethazine (Pharmaceutical Plant EGIS, Hungary), chlorpromazine (OOO Valenta Pharm, Russia), ipidacrine (OOO Ellara, Russia), chloral hydrate (Acros Organics, Belgium), Zoletil (Vibrac, France), medetomidine (OOO Apicenna, Russia), tiapride (AO Organica, Russia), droperidol (Moscow Endocrine Plant, Russia), diphenhydramine (Belkaroline, Belarus), and water for injection (Moscow Endocrine Plant, Russia) as well as 20% DMSO solution (AO Tatchimpharm medicines, Russia), administered in a volume of 0.5 mL. For each drug, 7–10 recordings were made on different animals. A new drug was administered until at least 3 days after the previous recording, to avoid interactions and residual effects. The number of tests (typically no more than 5–6) for each rat was determined based on the safety of the headplug (connector with electrodes) and the general condition of the animal. If there were signs of an infectious disease, Bicillin-3 (manufactured by OAO Syntez, Russia; at a dosage of 5000 U/kg, administered subcutaneously) would be re-injected and testing would not resume until at least one week after the injection. In case the animal’s condition did not improve, it was removed from the experiment. Another criterion for exclusion was a decrease in the quality of the background ECoG signal, which typically occurs when the animal has been tested for a prolonged period of time (more than one month). The most common signal deterioration is characterised by a significant decrease in amplitude across all channels or the emergence of epileptic activity (spikes and sharp waves in the ECoG recording). A total of 112 rats were included in the final analysis.

**Table 1:** Agents and their doses used as a training and validation set for compound 33a effects classification

**TRAINING SET**			**VALIDATION SET**		
**Agent**	**N**	**Targets**	**Agents**	**N**	**Targets**
Dizocilpine 0.25 mg/kg (DIZ 0.25)	9	NMDA	Promethazine 0.1 mg/kg (PMZ 0.1)	9	H1/ M-ChR/D2/D3/5-HT2_A_
Dexmedetomidine 0.005 mg/kg (DEX 0.005)	10	α2	Promethazine 0.5 mg/kg (PMZ 0.5)	10	
			Promethazine 5.0 mg/kg (PMZ 5.0)	10	
Dexmedetomidine 0.01 mg/kg (DEX 0.01)	10				
			Promethazine 20.0 mg/kg (PMZ 20.0)	10	
Galantamine 1.0 mg/kg (GAL 1.0)	10	AChE	Chlorpromazine 0.1 mg/kg (AMZ 0.1)	10	H1/ M-ChR/D2/D3/5- HT2_A_
Galantamine 3.0 mg/kg (GAL 3.0)	10				
			Chlorpromazine 1.0 mg/kg (AMZ 1.0)	10	
Haloperidol 0.3 mg/kg (HAL 0.3)	10	D2/D3			
Haloperidol 2.0 mg/kg (HAL 2.0)	10		Chlorpromazine 10.0 mg/kg (AMZ 10.0)	10	
Hydroxyzine 1.0 mg/kg (HXZ 1.0)	10	H1/5- HT2_A_	Ipidacrine 10.0 mg/kg (IPI 10.0)	9	AChE
Hydroxyzine 5.0 mg/kg (HXZ 5.0)	10		Ipidacrine 30.0 mg/kg (IPI 30.0)	10	
Hydroxyzine 20.0 mg/kg (HXZ 20.0)	10		Chloral hydrate 400.0 mg/kg (CHD 400.0)	10	GABA_A_
Sulpiride 30.0 mg/kg (SLP 30.0)	10	D2/D3	Tiletamine-zolazepam (ZOL/TLT)	9	NMDA/ GABA_A_
Sulpiride 100.0 mg/kg (SLP 100.0)	10		Medetomidine 0.8 mg/kg (MED 0.8)	9	α2
Tropicamide 0.5 mg/kg (TRO 0.5)	10	M-ChR	Tiapride 30.0 mg/kg (TIA 30.0)	10	D2/D3
Tropicamide 5.0 mg/kg (TRO 5.0)	10				
			Tiapride 100.0 mg/kg (TIA 100.0)	7	
Tropicamide 30.0 mg/kg (TRO 30.0)	10				
			Droperidol 0.3 mg/kg (DRO 0.3)	10	D2/D3
Phenazepam 0.1 mg/kg (PHE 0.1)	10	GABA_A_	Diphenhydramine 20 mg/kg (DPH 20.0)	10	H1/ M-ChR
Phenazepam 1.0 mg/kg (PHE 1.0)	10		H_2_O 0.5 mL	10	none
Aminophenylbutyric acid 100.0 mg/kg (PHB 100.0)	9	GABA_A_ / GABA_B_	DMSO 20% 0.5 mL	10	none
NaCl	10	none	TEST SET		
			33а – 50.0, 100.0 and 300.0 mg/kg		

###  ECoG signal analysis, dimensionality reduction and prediction

**Figure 1:**
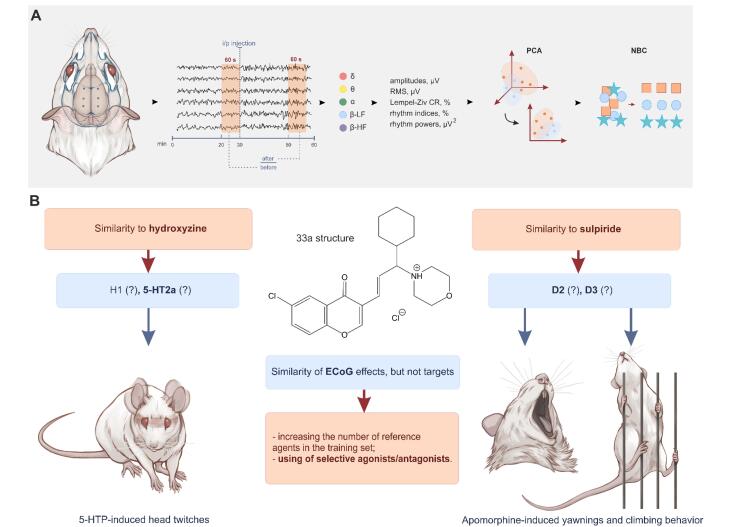
(A) Stages of the experiment; (B) Selected pharmacological tests to validate the results of the classification of compound 33а effects. I/p: intraperitoneal, RMS: root mean square, CR: compression ratio, LF: low-frequency, HF: high-frequency, PCA: principal component analysis, NBC: Naïve Bayes classifier, Ref: reference electrode

 The recordings obtained were analysed using the Neuron-Spectrum.NETomega 1.6.10.8 software (OOO Neurosoft, Russia). For all six channels (FP1, FP2, C3, C4, O1 and O2), 132 amplitude-spectral characteristics of the ECoG signal were calculated, including the mean and maximum amplitudes, the standard deviation of the amplitudes, and the Lempel – Ziv compression ratio. The last one characterizes the repeatability of the signal: the greater the repeatability, the higher the compression ratio. This indicator is embedded in many biomedical signal processing software packages and is used by some researchers to analyse EEG signals in rats.^15^ The ECoG signal was converted from the time domain to the frequency domain using the fast Fourier transform. Four different rhythms were extracted from the signal: δ- (0.5–4.0 Hz), θ- (4.0–8.0 Hz), α- (8.0–14.0 Hz) and β- (low-frequency (LF) — 14.0–20.0 Hz and high-frequency (HF) — 20.0–35.0 Hz). Mean amplitudes, indices, and mean power for each rhythm were calculated. The rhythm index was calculated as the ratio of the area under the spectral plot in the specific rhythm frequency range to the total area under the spectral plot in the passband. The average power was determined by averaging the power of the spectrum within a given frequency range. Parameters used in the power calculations included a quantization frequency (500 Hz), an analysis epoch length (5 s), maximum overlap between epochs (30%), number of points for fast Fourier transform (2048). Data were expressed as a ratio of parameter values following drug administration to those before administration (Figure 1). In this work and in previous papers,^8,9^ we calculated only the amplitude-spectral characteristics of the signal. However, there is also a considerable amount of information contained in data from cross-correlation and coherence analysis, which we have not yet explored. According to our observations (data not published), cross-correlation and coherence calculated by Neuron-Spectrum.NETomega do not provide a logical picture when used for classification with PCA and NBC.

 The processing and subsequent analysis of the data were performed using the XLSTAT 2016.02.28451 add-on for MS Excel. The data dimensionality reduction was achieved through the use of principal component analysis (PCA). The PCA was applied to the standardised X_norm (X – mean(X)/std(X)) data. After that, the decomposition of the correlation matrix X_norm^T*X was carried out. PCA is a widely used method for data preprocessing in machine learning. It reduces the dimensionality or large datasets by transforming them into a set of principal components that contain most of the information in the original data. PCA does this by transforming potentially correlated variables into a smaller set of variables, called principal components (PCs).

 After performing PCA, we trained an NBC model, and then classification was performed using six new integrative parameters: PC1, PC2, PC3, PC4, PC5, and PC6, which describe 83.57% of the variance in the data. The effect matrixes of dizocilpine, haloperidol, sulpiride, tropicamide, hydroxyzine, galantamine, dexmedetomidine, aminophenylbutyric acid and phenazepam were used as a training set. NBC is a classification algorithm that uses probability to determine the category that a data point belongs to. It assumes that all features in the data are independent of each other. PCA, on the other hand, simplifies complex datasets by converting them into a smaller set of uncorrelated variables. When NBC and PCA are combined, they create a more efficient model with higher accuracy. In comparison to other classification methods, Naïve Bayes is considered a more straightforward option due to its relative ease of parameter estimation. As a result, it is often the first algorithm introduced in data science and machine learning courses. Unlike logistic regression, Naïve Bayes can be considered as a fast and effective classifier that shows significant accuracy when the assumption of conditional independence holds. Additionally, the naive assumption makes the calculations very fast and requires less computational power than more complex algorithms. The common applications of NBC include spam filtering, sentiment analysis (determining positive, negative or neutral sentiment expressed in text) and document categorization. Additionally, it is often used in biomedical research for risks assessment and disease diagnostics.^6,7^

###  Behavioural tests

 Apomorphine-induced climbing test was performed on mice using the facilities of the “Open Science” Research and Manufacturing Complex (Russia). The facility consisted of 12 individual cylinders with a vertical grate. Two experiments were conducted, one with low doses and one with high doses of 33a. The mice were intraperitoneally injected with compound 33a at doses of 20, 50 and 100 mg/kg (Experiment 1), and 200 and 300 mg/kg (Experiment 2), comparison drug or saline solution. They were then immediately placed in the cylinder. Haloperidol at a dose of 0.3 mg/kg and sulpiride at doses of 50 mg/kg (Experiment 1) and 100 mg/kg (Experiment 2) were used as comparison drugs. The injection volume was 0.2 mL. After 30 min, mice were subcutaneously injected with apomorphine at a dose of 2 mg/kg and a 31-minute behavioural video recording began. Climbing was assessed every 10 min after apomorphine injection for 1 min. The time when the animal stood on its hind paws, holding onto the grate with its front paws, or hung completely on the grate, holding on with all paws, was estimated.^16-18^

 The apomorphine-induced yawning test was performed on rats at different doses of the drug (0.032 and 0.1 mg/kg). The animals were placed in a transparent Plexiglas box with a vertically standing mirror on the opposite side of the video camera. The behaviour of five animals was recorded at the same time. The rats were injected intraperitoneally with compound 33a (20, 50, and 100 mg/kg), a comparison drug, or saline solution. Haloperidol (0.3 mg/kg) and sulpiride (50 mg/kg) were used as reference drugs. The injection volume was 0.2 mL. After 30 min, the rats were injected subcutaneously with apomorphine at a dose of 0.032 or 0.1 mg/kg and behavioural recordings were started for 1 hour. The number of yawns of each animal was counted.^19-21^

 5-HTP-induced head-twitch test was conducted on mice housed individually in cages without coverslips. The mice were injected intraperitoneally with compound 33a at doses of 10, 20, and 50 mg/kg, haloperidol at a dose of 0.25 mg/kg or saline solution. The volume of the injection was 0.3 mL. After 30 min, the mice were given serotonin precursor 5-HTP (5-hydroxytryptophan) at a dose of 300 mg/kg by intraperitoneal injection, and their behaviour was recorded for 1 hour. The total number of head twitches was then assessed. 5-HTP was dissolved in 0.06 mL of dimethyl sulfoxide, and brought to a final volume of 0.3 mL with water for injection.^22,23^

###  Statistical analysis

 Statistical analysis of the obtained data was carried out using GraphPad Prism 8.0.2 software package (GraphPad Software, USA). The data distribution was assessed using the Shapiro–Wilk W-test. In the event of a normal distribution, the significance of the differences was determined using ANOVA with Dunnett’s post hoc test; for non-normal distributions, a non-parametric Kruskal–Wallis test followed by Dunn’s post-hoc analysis was used. The numerical values shown in the figures are presented as the mean ± SEM.

## Results

###  Drug-induced ECoG changes in rats

**Figure 2:**
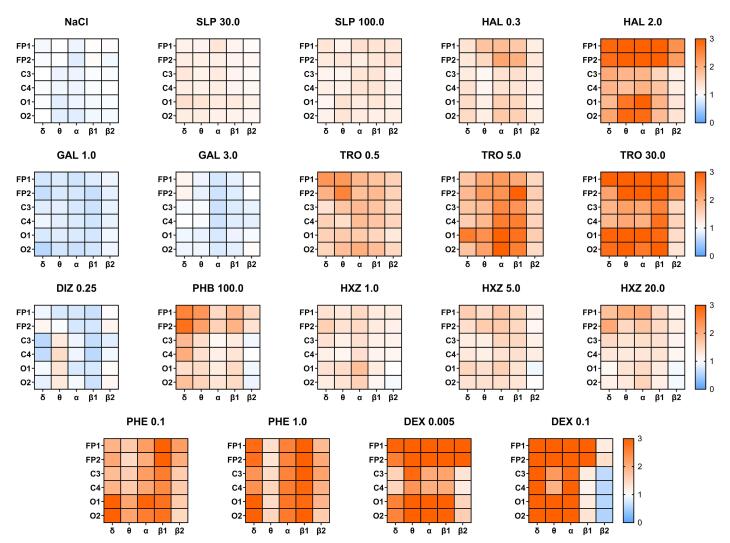
Heatmaps of median rhythm powers for the δ-, θ-, α- and β-rhythms in channels FP1, FP2, C3, C4, O1, and O2 following administration of saline solution (NaCl), sulpiride (SLP), haloperidol (HAL), galantamine (GAL), tropicamide (TRO), dizocilpine (DIZ), aminophenylbutyric acid (PHB), hydroxyzine (HXZ), phenazepam (PHE) and dexmedetomidine (DEX). The heatmaps reflect the ratios of mean rhythm power values at 20 min following drug administration to the respective values before the administration (~the 30th minute into the baseline record); 9–10 records per group were made

 Administration of drugs from the training set led to specific changes in the amplitude-spectral characteristics of ECoG in rats. Figure 2 shows heat maps of the median power values of the mean δ-, θ-, α- and β-rhythms recorded in the channels during administration of saline solution, sulpiride, haloperidol, galantamine, tropicamide, dizocilpine, aminophenylbutyric acid, hydroxyzine, phenazepam, and dexmedetomidine.

 The saline solution did not cause any significant changes in ECoG recorded in any of the channels or frequency ranges. Administration of sulpiride at a dose of 30 mg/kg resulted in a mild and uniform increase in mean power across all channels. When the dose was increased to 100 mg/kg, the effect was more pronounced. Haloperidol at a dose of 0.3 mg/kg also showed an increase in signal power, with a greater effect observed in channels FP1 and FP2 within the α- and β-LF wavebands. As the dose was raised to 2 mg/kg, the impact became more pronounced, particularly in frontal channels across all recorded frequency bands, as well as in O1 and O2 channels in the θ- and α-rhythm bands.

 Administration of galantamine, at a dose of 1 mg/kg, decreased the mean power of δ-, θ-, α- and β-rhythms in all channels. As the dose was increased to 3 mg/kg, the effect was maintained in both the α- and β-LF bands. Tropicamide, at a dose of 0.5 mg/kg, resulted in an increase in power in all frequency channels, with a more pronounced effect at low frequencies in frontal channels. This drug, when administered at a dose of 5 mg/kg, significantly increased the mean power of the α- and β-HF rhythms, with predominance in occipital channels. This effect was more pronounced when the dose was 30 mg/kg, although it was not observed in the β-HF band and parietal channels. Dizocilpine, at a dose of 0.25 mg/kg, had a mixed effect. On the one hand, it decreased mean power in most bands, especially in the β-LF. On the other hand, θ-rhythm power increased in the parietal and occipital channels. Aminophenylbutyric acid, at a dose of 100 mg/kg, increased all-band power in the FP1 and FP2 channels, especially at low frequencies. However, mean β-HF band power decreased in C3, C4, O1 and O2 channels. Hydroxyzine, at a dose of 1 mg/kg, moderately increased the power of the rhythms, especially in the α-band. When the dose was increased to 5 mg/kg, there was a more significant increase in power in the α- and β-NF bands. At 20 mg/kg, the drug had a similar effect, but it was more pronounced in frontal channels. Phenazepam, at a dose of 0.1 mg/kg, caused a significant increase in the spectral power of all rhythms, especially in the occipital channels and in β-NF band in all channels. When the dose was increased to 1 mg/kg, the effect continued to increase, but it did not affect θ-frequencies. There were a significant increase in δ-rhythm power, however. Dexmedetomidine, at a dose of 0.005 mg/kg, dramatically increased power over the entire frequency range in frontal channels. Power in channels O1 and O2 increased at all frequencies, except for β1. Power also increased in channels C3 and C4 across the entire frequency range, except for δ- and β-NF. At a dose of 0.1 mg/kg, there was a pronounced increase in the mean power of δ-, θ- and α-bands in all channels. However, in the β-HF band, the increase was only observed in FP1 and FP2 channels, while power decreased in parietal and occipital channels.

###  Data dimensionality reduction using principal component analysis

**Figure 3:**
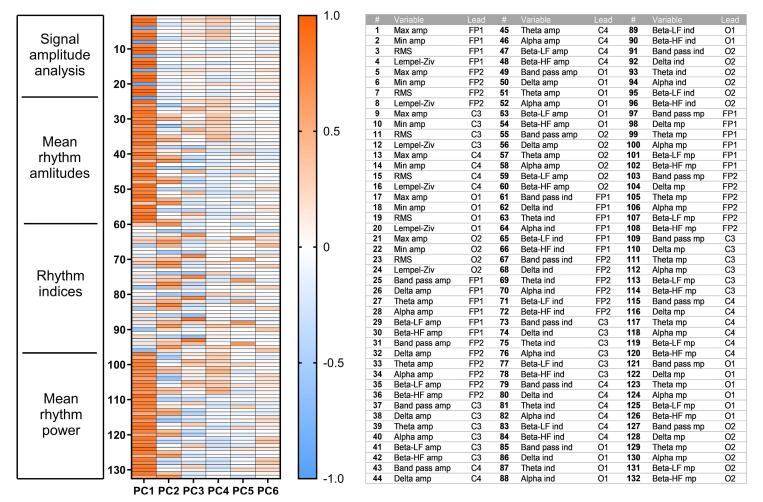
Factor loadings reflecting the degree of involvement of each of the 132 parameters analysed into the formation of the PC1, PC2, PC3, PC4, PC5 and PC6 components, used for the subsequent NBC analysis. Amp: amplitude (μV), RMS: root mean square (μV), Lempel-Ziv: Lempel – Ziv compression ratio (%), ind: rhythm index (%), mp: mean rhythm power (μV^2^)

 Analysis of the obtained PCA data showed that 83.57% of the variance could be explained by six principal components (PC1 to PC6), which were used for further analysis. The factor loadings of the amplitude-spectral characteristics of ECoG, describing their contribution to the formation of one or another principal component, are presented in a heatmap (Figure 3). PC1, accounting for 49.1% of the total variance, is formed by amplitude features of the signal, both as a whole signal or individual rhythms, as well as correlated values of the spectral power of the rhythms. All these ECoG parameters contribute to PC1 regardless of electrode location. PC2 (14.0%), on the other hand, is more influenced by δ- and β-rhythms activity in all channels. PC3 (9.6%) is almost entirely determined by δ- and θ-rhythmic indices. The PC4 component (4.5%) was formed predominantly by the magnitude of the mean amplitude and power of the θ-rhythm in parietal and occipital cortical areas. The values of PC5 (3.9%) depended on the mean α-rhythm power in channels C3, C4, O1, and O2. The PC6 component (2.4%) was determined by the values of β-HF rhythm index as well as mean δ-rhythm power in occipital areas O1 and O2.

 Next, values of the six new parameters (principal components PC1–PC6) were calculated for all records from the training set as well as the three doses of 33a (Supplementary file 1). Table 2 shows examples of PC values of ten records of the compound studied at a dose of 300 mg/kg.

**Table 2:** Example of calculated PC1–PC6 values for ten records obtained after administration of 33a at a dose of 300 mg/kg

**Record number**	**Principal components**					
	**PC1 (49.1%)**	**PC2 (14.0%)**	**PC3 (9.6%)**	**PC4 (4.5%)**	**PC5 (3.9%)**	**PC6 (2.4%)**
1 (#63)	–2.56	–1.85	2.43	1.89	–1.29	–1.47
2 (#68)	–7.00	2.16	1.76	–0.46	–0.27	–0.04
3 (#70)	0.14	1.06	1.58	0.77	0.20	–0.93
4 (#71)	–5.43	1.33	–1.02	1.09	–0.83	–0.39
5 (#75)	–4.78	2.76	2.01	–1.67	–0.25	–0.04
6 (#77)	–3.66	0.98	4.80	–2.03	–0.69	–0.19
7 (#78)	–4.75	–2.15	1.36	–0.21	2.26	0.17
8 (#79)	–6.14	1.79	0.20	0.76	1.08	–0.58
9 (#80)	–1.70	–1.04	2.68	–0.48	0.76	–1.34
10 (#81)	–4.97	1.74	3.39	–1.85	–1.81	–1.39

*Note:* the percentage of variance in the data described by the corresponding principal component is given in parentheses next to the principal components; the number of the corresponding animal is given in parenthesis opposite the recording number.

**Figure 4:**
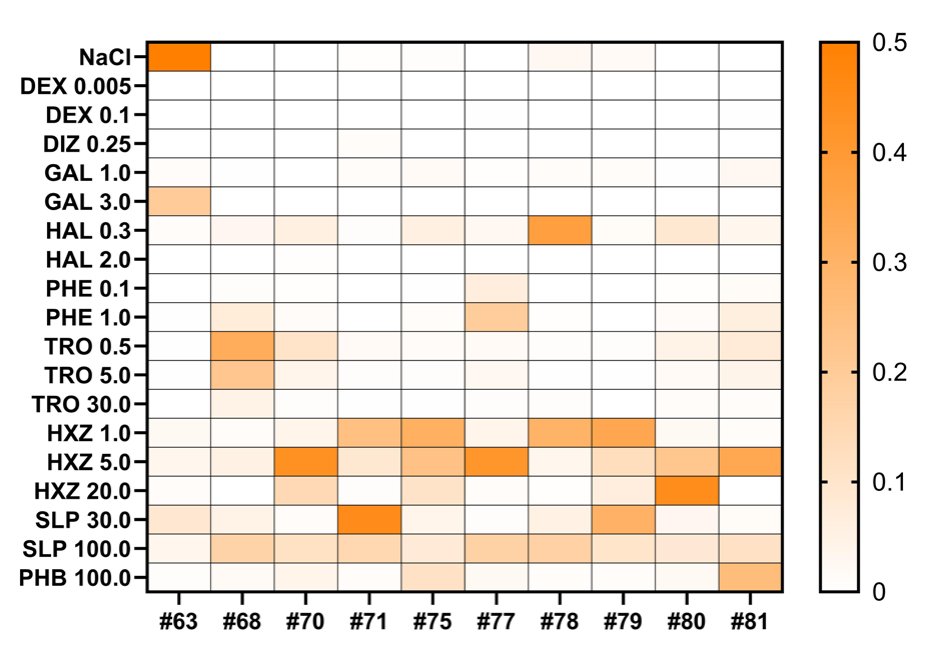
Example of calculated probability values for the probability of matching the ECoG effects of 33a at a dose of 300 mg/kg in 10 rats (#63, #70, #71, #75, #77, #78, #79, #80, #81, #86) with the effects of training set drugs

**Figure 5:**
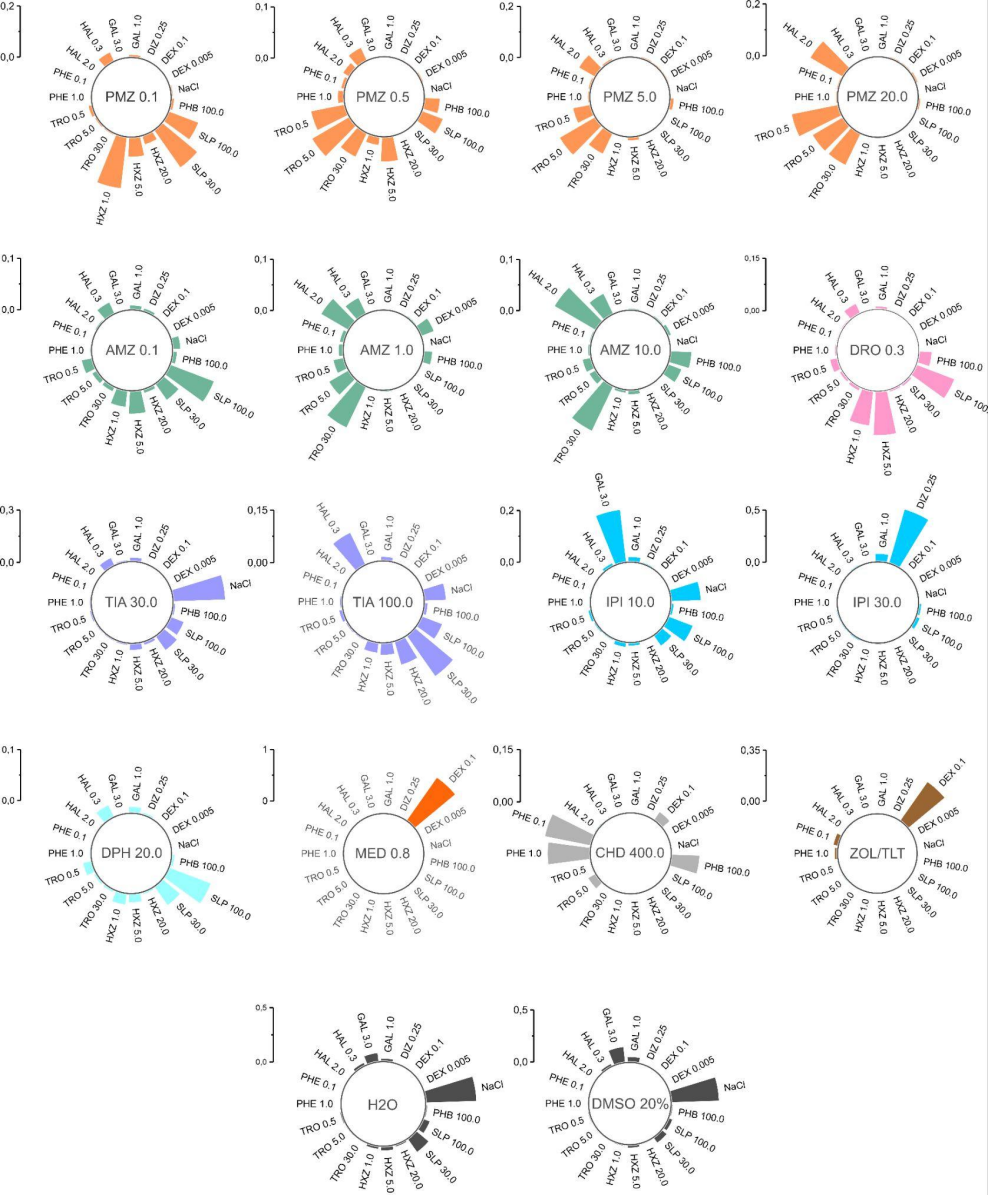
Radial charts of the calculated median probability of similarity values of the drugs from the validation set group to the drugs in the training set obtained using NBC. PMZ: promethazine, AMZ: chlorpromazine, DRO: droperidol, TIA: tiapride, IPI: ipidacrine, CHD: chloral hydrate, DPH: diphenhydramine, ZOL/TLT: zolazepam/tiletamine

 The pharmacological activity of the validation set of drugs, as well as compound 33a, was classified at doses of 50, 100, and 300 mg/kg using the NBC method based on the calculated data. For each record, the probabilities of ECoG effects matching those of the training set drugs were obtained: saline solution, sulpiride, haloperidol, galantamine, tropicamide, dizocilpine, aminophenylbutyric acid, hydroxyzine, phenazepam, and dexmedetomidine (Figure 4). Next, the median value of the probability of similarity (MPS) to a reference group was calculated for all tested groups, from which conclusions could be drawn about the nature of action of a particular agent from the validation set (Figure 4, Supplementary file 2) or compound 33a (Figure 5, Supplementary file 2).

###  Classification of ECoG effects of the validation set and 33a

 The antiemetic drug promethazine (PMZ) at the lowest dose of 0.1 mg/kg showed marked similarity to the dopamine blocker sulpiride at doses of 30 and 100 mg/kg (MPS = 0.175 and 119, respectively) and the histamine blocker hydroxyzine at doses of 1 mg/kg (0.198) and 5 mg/kg (0.073). With increasing dose of promethazine, the similarity shifted towards the cholinolytic agent tropicamide: for promethazine at a dose of 0.5 mg/kg, the MPS was 0.064, 0.082, and 0.049 with tropicamide at doses of 0.5, 5, and 30 mg/kg, respectively. When the dose of promethazine was increased to 5 mg/kg, only tropicamide remained clearly similar, particularly at the 5 mg/kg dose (0.155). The highest dose of promethazine, 20 mg/kg, also showed tropicamide-like effects (MPS = 0.179, 0.131, and 0.130 for doses of 0.5, 5, and 30 mg/kg, respectively), but also showed similarities to the high dose of haloperidol 2 mg/kg (0.142).

 The effects of the antipsychotic chlorpromazine (AMZ) at a dose of 0.1 mg/kg were similar to those of sulpiride 100 mg/kg (0.088) and hydroxyzine 5 mg/kg (0.042). As the drug dose was increased to 1 mg/kg, a predominant similarity to tropicamide 30 mg/kg appeared (0.086). The 10 mg/kg dose had the highest similarity to haloperidol 2 mg/kg (0.091) and tropicamide at the highest dose of 30 mg/kg (0.094). A typical neuroleptic from the butyrophenone group, droperidol (DRO) at a dose of 0.3 mg/kg, was more similar in its effects to sulpiride 100 mg/kg (0.113) and hydroxyzine 5 mg/kg (0.123). The atypical neuroleptic tiapride (TIA) at a dose of 30 mg/kg had effects similar to saline solution (0.290) and, to a lesser extent, sulpiride at doses of 30 and 100 mg/kg (0.192 and 0.074, respectively). At a dose of 100 mg/kg, tiapride exhibited more pronounced effects on ECoG in rats and was determined as sulpiride at a dose of 30 mg/kg (0.138), haloperidol 0.3 mg/kg (0.098) and hydroxyzine 20 mg/kg (0.066).

 The AChE inhibitor ipidacrine (IPI) at a dose of 10 mg/kg showed similarity to galantamine at a dose of 3 mg/kg (0.200) and saline (0.111). At a dose of 30 mg/kg, the drug was more close to dizocilpine 0.25 mg/kg (0.526) and galantamine 1 mg/kg (0.066). The antihistamine drug diphenhydramine (DPH) at a dose of 20 mg/kg showed preferential similarity to sulpiride at doses of 30 and 100 mg/kg (0.042 and 0.084, respectively). The adrenomimetic medetomidine (MED) at a dose of 0.8 mg/kg was defined exclusively as dexmedetomidine at a dose of 0.1 mg/kg (1,000) by the classifier. The general anaesthetic chloral hydrate (CHD) 400 mg/kg was classified as phenazepam 1.0 and 0.5 mg/kg (0.132 and 0.119, respectively) and phenibut (0.078). Another anaesthetic drug, Zoletil, which is a combination of tiletamine and zolazepam, (ZOL/TLT) was also identified as dexmedetomidine 1.0 mg/kg (0.319). Water for injection (H_2_O) and 20% aqueous dimethyl sulfoxide (DMSO) solution were both defined as NaCl (0.454 and 0.440, respectively).

**Figure 6:**
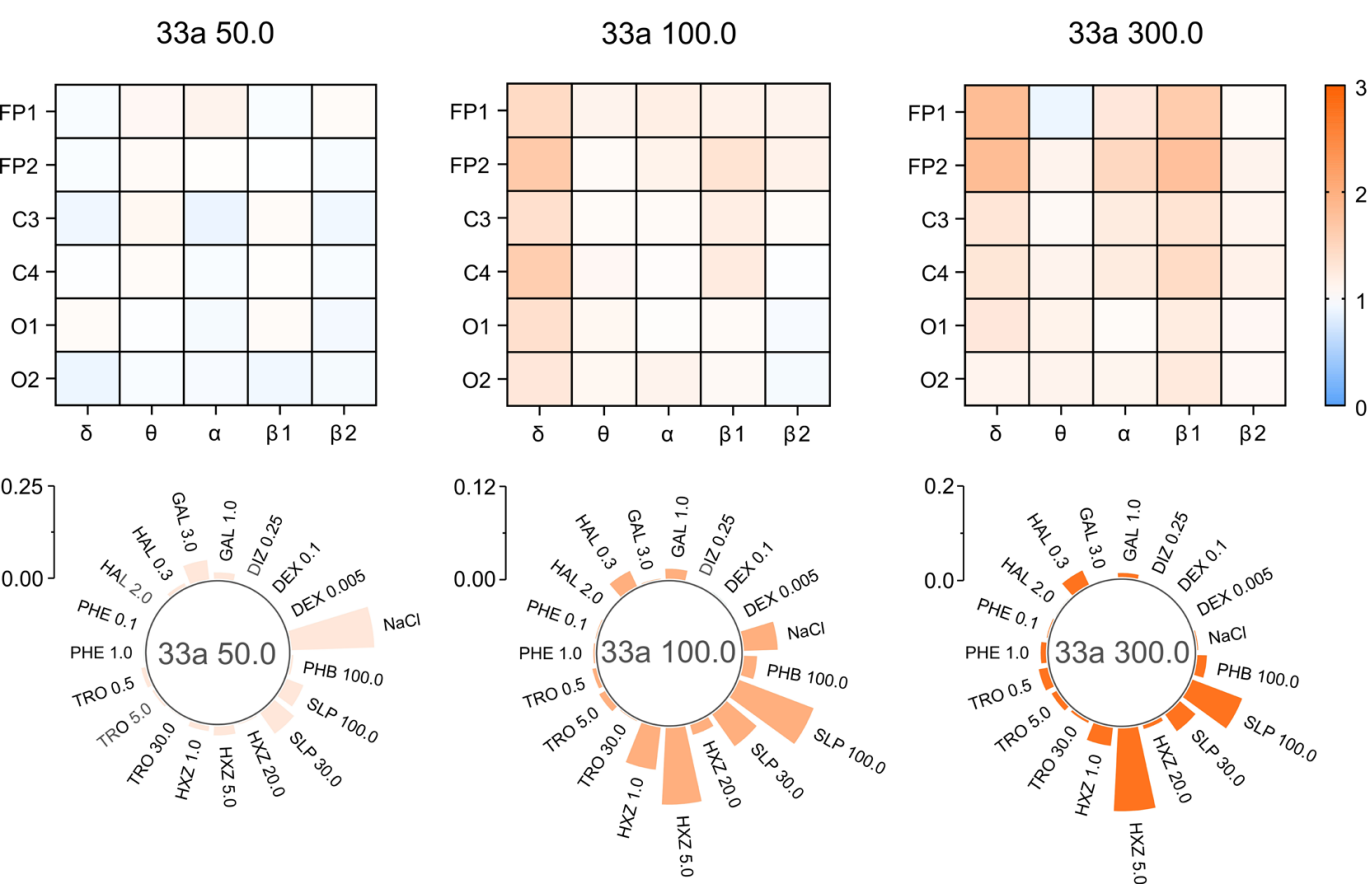
Radial charts of the calculated median probability of similarity values of three doses of 33a to the training set drugs obtained using NBC

 Compound 33a at a dose of 50 mg/kg showed no marked similarity to any of the drugs in the training set, resulting in ECoG changes similar to those following saline administration (MPS = 0.224). However, when the dose was increased to 100 mg/kg, similarities with sulpiride 100 mg/kg (0.102) and hydroxyzine 5 mg/kg (0.098) became more evident. At a dose of 300 mg/kg, 33a showed the strongest similarity to hydroxyzine 5 mg/kg (0.175) and sulpiride 100 mg/kg (0.112). This dose also showed some similarity to another dopamine antagonist, haloperidol, at a dose of 0.3 mg/kg (0.033) (Figure 6).

 Thus, it is found that compound 33a at high doses shows similarity to drugs with H1-histamine-, 5-HT2a-blocking- and dopamine-blocking effects.

###  Behavioural testing of 33a

**Figure 7:**
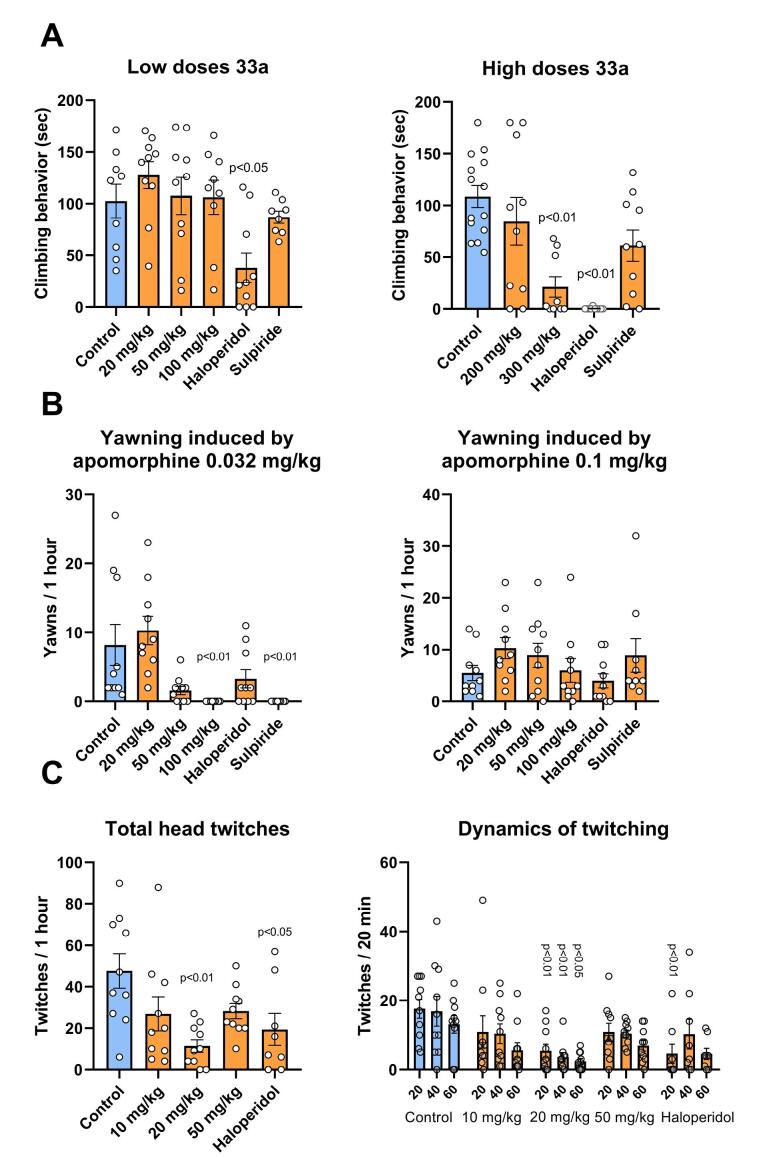
Results of behavioural tests: (A) ‘Apomorphine-induced climbing’ with low (left) and high (right) doses of compound 33a. (B) ‘Apomorphine-induced yawning’ with low (left) and high (right) doses of apomorphine. (C) ‘5-HTP-induced head-twitch test’ with total number of head-twitches (left) and temporal dynamics of the effect (right). Haloperidol was administered at a dose of 0.3 mg/kg except for the apomorphine-induced climbing test, where the dose was 0.25 mg/kg. Sulpiride was administered at a dose of 50 mg/kg with the exception of the apomorphine-induced climbing test (high dose 33a) where the dose of sulpiride was 100 mg/kg. In the control group, saline solution was administered. *P* < 0.05, *P* < 0.01 vs. controls, Dunnett’s post-hoc test for significant ANOVA data, or Dunn’s post-hoc test for significant Kruskal–Wallis data

 To validate the classification results obtained, behavioural tests were conducted, including two tests on mice: the Apomorphine-induced Climbing and 5-HTP-induced Head-twitch Test, and one test on rats: the Apomorphine-induced Yawning (Figure 7).

 The apomorphine-induced climbing test was performed in two stages. In the first stage, low doses of compound 33a were tested (20, 50 and 100 mg/kg), as shown in Figure 7A (left). In the second stage, high doses (200 and 300 mg/kg) were administered (Figure 7A, right). When the compound was given at doses between 20 and 200 mg/kg, there was no difference in climbing time compared to the control group. However, when 33a was administered at a dose of 300 mg/kg, it resulted in a significant decrease in total climbing time, to 21 ± 10 s compared to 108 ± 11 s in the control group (*P* < 0.01; Dunn’s criterion, Supplementary file 3). It is worth noting that the administration of 33a at a dose of 300 mg/kg resulted in the death of four out of ten animals within a day of testing. However, the comparison drug haloperidol, at a dose of 0.3 mg/kg, significantly reduced the total climbing time to 38 ± 14 s (*P* < 0.05; Dunn’s criterion, Supplementary file 3) in the low-dose 33a test, and to 0.3 ± 0.3 s (*P* < 0.01; Dunn’s criterion, Supplementary file 3) in the high-dose 33a test. Sulpiride did not have a significant impact on the effects of apomorphine, either at the 50 or 100 mg/kg dose.

 Two doses of apomorphine were used in the apomorphine-induced yawning test: 0.032 mg/kg (Figure 7B, left) and 0.1 mg/kg (Figure 7B, right). In a test with a 0.032 mg/kg dose of apomorphine, the administration of compound 33a at 100 mg/kg completely eliminated yawning, as did the administration of sulpiride at 50 mg/kg (*P* < 0.01; Dunn’s criterion, Supplementary file 3). Another reference drug, haloperidol, also reduced the number of yawns, but these effects were not statistically significant. At an apomorphine dose of 0.1 mg/kg, no differences were found with the control in either group.

 Administration of 33a at a dose of 20 mg/kg in the 5-HTP-induced head-twitch test resulted in a reduction in the total number of head twitches to 11 ± 3 compared to 47 ± 8 in the control group (*P* < 0.01; Dunnett’s criterion, Supplementary file 3). Similarly, administration of haloperidol at a dose of 0.3 mg/kg also reduced head twitches to 19 ± 8 (*P* < 0.05; Dunnett’s criterion, Supplementary file 3). The effect of compound 33a at a dose of 20 mg/kg was also maintained when the temporal dynamics of head twitches was analysed in more detail. In the first 20 minutes, the number of head twitches decreased to 5 ± 2 vs. 17.6 ± 2.7 in the control (*P* < 0.01; Dunnett’s criterion, Supplementary file 3). In the second 20-minute period, the number of head twitches decreased to 3.6 ± 1.3 vs. 16.9 ± 4.3 in the control (*P* < 0.01; Dunnett’s criterion, Supplementary file 3). After the third 20-minute period, the number of head twitches decreased to 2.4 ± 0.8 vs. 13.1 ± 2.5 (*P* < 0.05; Dunnett’s criterion, Supplementary file 3). Administration of haloperidol at a dose of 0.3 mg/kg reduced the number of head twitches in the first 20 minutes of the test to 4.6 ± 2.8 (*P* < 0.01; Dunnett’s criterion, Supplementary file 3).

## Discussion

 In the conducted study, we found that PCA combined with NBC can detect the effects of several groups of psychoactive drugs on ECoG. These drugs include blockers of D2/D3-, M-choline-, and H1-histamine receptors, as well as AChE inhibitors and agonists of GABA- and alpha-2-adrenoreceptors. For promethazine and chlorpromazine, we observed a dose-dependent shift from similarity with sulpiride (a D2/D3-blocker) and hydroxyzine (an H1- and 5-HT2a-receptor blocker) to tropicamide- and haloperidol-like actions (M-choline-blocking and dopamine-blocking, respectively). In contrast to previous work,^9^ in the present study, these two drugs showed a closer similarity to the reference histamine-blocking agents, hydroxyzine, used primarily as an anti-anxiety agent,^24^ instead of chloropyramine, which does not cross the blood-brain barrier well (as indirectly evidenced by its ECoG effects). It is important to note that the high dose of chlorpromazine (10 mg/kg) showed similarity not to the antipsychotic dose of haloperidol (0.3 mg/kg) but to the cataleptogenic dose (2.0 mg/kg), which may suggest that this dose is “cataleptogenic”, and which is consistent with the results of other authors.^25^ Two other antipsychotics from the validation set, droperidol and tiapride, showed similarity to haloperidol, sulpiride, and hydroxyzine, which is an expected result.

 The classification of chloral hydrate and medetomidine was the most accurate, resembling phenazepam and dexmedetomidine, respectively. This indicates that agents with direct GABAergic and alpha-2-adrenomimetic actions produce the most specific ECoG effects, which allows to clearly classify them using NBC. The combination of the GABA-ergic zolazepam and the NMDA antagonist tiletamine (Zoletil 100) was rated by the classifier as ‘dexmedetomidine’, which on the one hand is fair, as both agents are general anaesthesia drugs. However, it is fair to question why there was no similarity to phenazepam (as well as zolazepam, a direct GABA mimetic) and at least some similarity to dizocilpine. Perhaps, in the first case, this is due to the insufficient sensitivity of the proposed method, either in terms of registration of brain bioelectrical activity, or in terms of the classification algorithm. The lack of similarity with dizocilpine could be explained by the fact that zolazepam may ‘overlap’ the ECoG effects of tiletamine, preventing the identification of its specific effects. This assumption is supported by the fact that the drug is rarely used for recreational purposes,^26^ unlike other NMDA receptor antagonists such as phencyclidine and ketamine.^27,28^ In future studies, it would be beneficial to include either the unmixed tiletamine or the dissociative anaesthetic ketamine in the validation set in order to more accurately validate the detection of NMDA-blocking effects. The H20 and DMSO 20% groups demonstrated clear similarities with the saline group, indicating that the proposed method can detect the absence of a pharmacological effect per se.

 Less “successful” classifications include the prediction results for ipidacrine and diphenhydramine. The high dose of ipidacrine was classified as “dizocilpine”. The NMDA-blocking action for this drug seems unlikely, and the result obtained may be, as in the case of Zoletil-dexmedetomidine, due to similarities in effects but not in molecular targets. Both ipidacrine at a dose of 30 mg/kg and dizocilpine induced excitation with hyperactivity, which resulted in increased power of theta rhythms in channels C3, C4, O1 and O2, even when motion artifacts are excluded. It is probably this feature of the ECoG that influenced the classifier in such a way that the two drugs were identified as similar, ignoring the differences in their molecular targets of action. Despite this, ipidacrine at 10 mg/kg showed high similarity to another AChE inhibitor, galantamine, indicating a certain sensitivity of the method to the effects of this group of drugs.

 Diphenhydramine, despite expected similarities with hydroxyzine and tropicamide, has been classified more as a sulpiride. Although no data on its affinity for D2- and D3-receptors were found in the literature, a possible dopamine-blocking action (as well as a deficiency in classifier performance) cannot be completely ruled out. H1-histamine receptor blockers are among the most challenging groups to implement the proposed screening method for. Since most first-generation drugs (chloropyramine, diphenhydramine, etc.) have strong M-cholinergic-blocking effects,^29^ they are not suitable for use in the training set. Second and third-generation blockers, despite being selective to H1 receptors, have difficulty penetrating the blood-brain barrier^30^ and therefore cannot produce significant effects on ECoG. Intraventricular injection of selective agents via a cannula may offer a potential solution, but this approach introduces additional variables into the experiment and increases the risk of infection. In general, despite some limitations in the classifier’s performance, the results obtained give us reason to be optimistic about the further development of the method. To further improve its efficiency, we need to increase the training and validation sets by adding more reference psychotropic drugs, as well as to optimize the settings of the classification algorithms used.^1^

 In this study, the pharmacological screening of molecule 33a was performed for the first time using pharmaco-EEG and machine learning techniques (NBC). Our approach revealed that the molecule has dose-dependent similarity with hydroxyzine and sulpiride, which may indicate H1-histamine-, 5-HT2a-blocking as well as anti-dopaminergic actions. To validate the results, we conducted classical pharmacological tests to detect 5-HT2a- and dopamine-blocking effects. The drug abolished the effects of low-dose apomorphine in rats and high-dose 5-HTP in mice, confirming the predicted activities of the studied compound.

 The blockade of 5-HT2a receptors may determine the antianxiety and antidepressant effects of some antidepressants and atypical neuroleptics.^31^ This mechanism may explain, to some extent, the anxiolytic effect of 33a in zebrafish in the novel tank test and the light/dark box test.^11^ However, later experiments on the BALB/c mice strain did not show a statistically significant decrease in the level of anxiety.^12^ This could probably be due to the fact that the 20 mg/kg dose, which was effective in the 5-HTP test in the present study, was not tested. In the BALB/c mice strain, 33a at a dose of 10 mg/kg increased the frequency and time spent in the open arms and the number of head dips over the edge of the open arms in the elevated plus maze test. However, these behavioural changes were not found to be statistically significant. A higher dose of 50 mg/kg was tested next, and it reduced locomotor activity in the elevated plus maze test (but not in the Open Field), possibly due to the involvement of additional mechanisms that masked a potential anti-anxiety effect.

 To evaluate the potential dopamine-blocking activity of compound 33a, we performed classical tests such as apomorphine-induced climbing behaviour and apomorphine-induced yawning.^32^ In the former test 33a blocked stereotypy only at a high dose (close to LD_50_), which may indicate low activity against dopamine receptors. This conclusion may be premature, however, considering the work by Sukhanov et al,^33^ who reported that some behavioural effects of apomorphine, particularly climbing behaviour, are associated with the activation of TAAR1 receptor. In contrast, the presence of a dopamine-blocking effect of 33a is supported by the results of the second test where apomorphine triggered another form of rat behaviour, yawning. A series of classical neuropharmacological studies by Collins et al^19,20^ using selective agonists and antagonists of dopamine receptors, demonstrated that this form of behaviour is associated with the activation of the D3 receptor subtype. At the same time, when the dose of dopamine mimetics such as apomorphine, 7-OH-DPAT, PD-128,907, etc. is increased, D2 receptors are activated, resulting in decreased yawning behaviour. In this regard, the yawning induced by dopamine receptor agonists has a U-shaped dose-effect curve. In our study, compound 33a at a dose of 100 mg/kg inhibited yawning induced by administration of apomorphine at a dose of 0.032 mg/kg. However, when the dose of apomorphine was increased to 0.1 mg/kg, the same dose of the studied substance was ineffective, and lower doses of 20 and 50 mg/kg not only failed to abolish the behavioural phenotype, but also increased its expression (although without statistical significance). This feature of the action may be related to the fact that 33a, at low doses, blocks D2 receptors, thereby increasing the number of yawns induced by apomorphine at a higher dose. However, increasing the dose of 33a results in the blockade of D3 receptors, which depresses the effects of lower doses of apomorphine. Interestingly, we observed similar dynamics when administering the comparison drug, sulpiride. A biphasic action has also been shown for haloperidol, in the work of Collins et al,^20^ in which yawning was induced by the dopaminomimetic PD-128,907.

**Figure 8:**
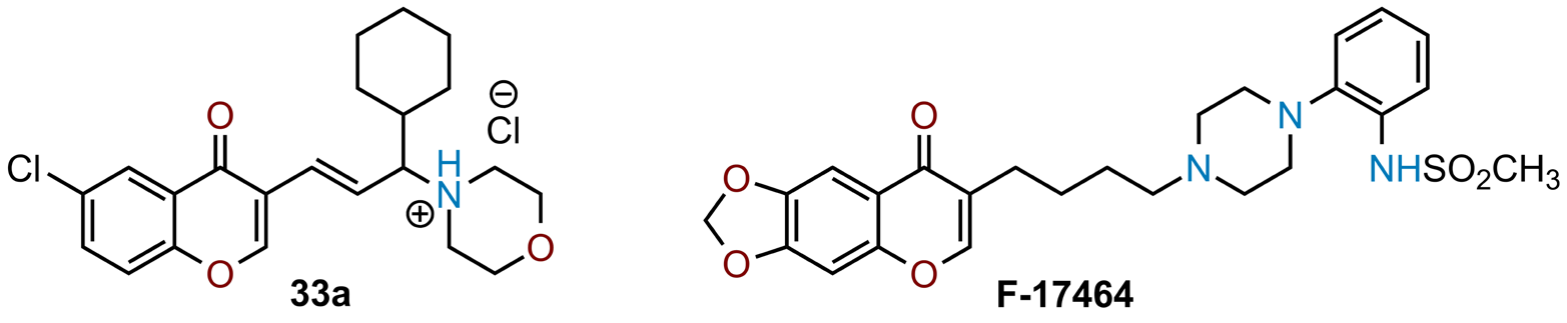
Chemical structure of chromone-containing compounds 33a and F-17464

 Some chromone-containing alkylamines, structurally similar to molecule 33a, have previously been described as psychotropic agents.^34,35^ In particular, 3-(4-piperazinobutyl)chromones, synthesized by Sokoloff et al. were shown to be ligands of dopamine D3 receptors.^34^ Additionally, compound F17464,^36^ which has successfully completed phase II clinical trials for the therapy of acute exacerbation of schizophrenia.^37,38^ did not cause weight gain or extrapyramidal symptoms in patients, although rare cases of akathisia were reported (Figure 8).

 Chernov et al. described more than 25 chromone-containing derivatives of allylmorpholine, for which inhibition of acetyl- and butyrylcholinesterase, as well as blocking action towards NMDA receptors were shown to varying degrees.^10^ In work with zebrafish, the simultaneous administration of NMDA receptor agonists (NMDA and quinolinic acid) and the M-cholinergic blocker biperiden failed to abolish the effects of compound 33a and other derivatives.^11^ In the present study, we found no similarity between the effects of compound 33a on the ECoG and those of the NMDA antagonist dizocilpine or the AChE inhibitor, galantamine, at any of the studied doses. When 33a was administered even at a high dose (300 mg/kg), we did not observe any lacrimation or salivation, which are generally classical effects of AChE inhibitors.^39^ For another molecule, 33b, an allylmorpholine derivative, Chernov et al.^10^ showed the highest activity towards targets *in vitro* (not inferior to memantine and donepezil) and therefore considered it a very promising neuroprotective agent. Nevertheless, in the traumatic brain injury model in rats, a 7-day course of treatment with 33b did not significantly affect the severity of the neurological deficit in injured animals.^40^ Based on the results of the previous and current studies, it can be concluded that the molecular targets of chromone-containing allylmorpholine derivatives, as demonstrated *in vitro*, may not be relevant *in vivo*.

 The present study has allowed, to some extent, to shed light on the effects and mechanisms behind the dose-dependent suppression of locomotor activity previously observed in zebrafish and mice.^11,12^ The combination of 5-HT2- and dopamine-blocking action allows 33a to be considered as a possible promising antipsychotic drug. For atypical neuroleptics, 5-HT2a-receptor blockers have been shown to increase dopamine release in the mesocortical and nigrostriatal systems, which has a beneficial effect on negative symptoms and reduces the risk of extrapyramidal side effects.^41^ In a recent meta-analysis^42^ of 10 studies involving 1,714 patients it was found that the use of 5-HT2A antagonists (ritanserin, pimavanserin, cyproheptadine, roluperidone, trazodone, and eplivanserin) in combination with neuroleptic medications or monotherapy significantly reduced negative symptoms in people with schizophrenia. The study also found that patients who received antiserotonin medications had fewer extrapyramidal side effects compared to those who received a placebo. Based on these findings, a more detailed study of the 33a molecule, as well as other chromone-containing allylmorpholine derivatives, seems promising. These agents should be tested using more specific behavioural tests, such as prepulse inhibition^43^ or active avoidance conditioning, due to high translational value.^44^ In addition, the effects of the drug on social interaction and cognitive functions in rodents seem to be of interest, which is particularly significant for drugs targeting affective disorders. Furthermore, since the drug demonstrated similar ECoG effects exactly with sulpiride but not with haloperidol, despite their common antiserotonin action, their targeted comparison warrants special attention. One of the main advantages of sulpiride in clinical practice is its moderately stimulating and antidepressant properties,^45^ but patients may experience increased prolactin levels,^46^ which can reduce their compliance.

 The experimental results presented in this study should be interpreted with caution, given some limitations. Firstly, it is important to note that the effectiveness of the proposed pharmaco-EEG screening technique depends on the size and quality of the training set. If the mode of action or effects of the compound under investigation differ from those of the drugs included in the training set, the NBC may make incorrect predictions. Therefore, it is possible that some effects other than dopamine- and 5-HT2-blocking may have been missed. For instance, our training set did not include agents that act on trace amines receptors, and the effect on these receptors cannot be excluded for compound 33a, if the obtained results allow it to be considered as a potential antipsychotic. Therefore, in the future, we plan to expand the training set with new reference drugs and, over time, new effects may be discovered for the studied compound. Additionally, it is possible that other machine learning algorithms may provide more accurate predictions when applied to the collected training sample. To address this question, further research is needed to compare different methods of data preprocessing and dimensionality reduction, justify the choice of the optimal number of final parameters, and compare various machine learning algorithms, such as NBC, logistic regression, support vector machine (SVM), random forest and others. Finally, pharmaco-EEG screening of other CCAMs is highly warranted. In the previous study,^11^ at least five of them demonstrated the ability to suppress the *Danio rerio* activity. It can be assumed that the results of their pharmaco-EEG screening will be similar. This comparison, on the one hand, will allow additional assessment of the validity of the proposed method and, on the other hand, it will help to establish a better understanding of the structure-activity relationship.

## Conclusion

 The present study has once again demonstrated the effectiveness of principal component analysis and the Naïve Bayes classifier combination in classifying the pharmacological activity of compounds based on their effects on the amplitude-spectral characteristics of ECoG in rats. The similarities of the effects of compound 33a with hydroxyzine and sulpiride, as well as the elimination of the effects of apomorphine and 5-hydroxytryptophan in mice and rats, suggest that the studied molecule possesses dopamine- and 5-HT2-blocking effects. The evaluation of potential H1-histamine-blocking activity was not performed in the present work, but is also of interest for further study. The results obtained suggest that compound 33a may have antipsychotic properties similar to those of atypical neuroleptics in terms of mechanism of action. Therefore, further validation of the compound through a larger number of experiments and translational models would be beneficial.

## Competing Interests

 The authors declare no conflict of interest.

## Data Availability Statement

 Not applicable.

## Ethical Approval

 Experiments using laboratory animals were performed in accordance with the requirements of Directive 2010/63/EU of the European Parliament and Council of September 22, 2010, the principles of the Basel Declaration and the requirements of the Council of the Eurasian Economic Union from November 14, 2023, No. 33 “On Guidelines for handling laboratory (experimental) animals when conducting preclinical (non-clinical) studies”. The protocol of the experiment was approved by the Bioethical Commission of Saint Petersburg State Chemical and Pharmaceutical University of the Ministry of Health of Russia (protocol-application R-PEEG2-SA-2022 dated February 15, 2022). All measures were taken to reduce the number of animals used and minimize their suffering.

## Informed Consent Statement

 Not applicable.

## Supplementary Files


Supplementary file 1. PC1-PC6 values for training, validation and test sets.

Supplementary file 2. Probability values of validation and test sets.

Supplementary file 3. Behavioural tests statistics.
