# Supplementary material for: Pharmaco-EEG-Based Classification of Psychotropic Activity of a Novel Chromone-Containing Allylmorpholine in Rats
Source: Adv Pharm Bull. 2025 Oct 11;15(4):891–905. doi: 10.34172/apb.025.45140 (PMC12980273; doi:10.34172/apb.025.45140)
Supplement: Supplementary file 3 — Behavioural tests statistics. [file apb-15-891-s003.pdf]

Adv Pharm Bull. 2025;15(4):891-S3
doi: 10.34172/apb.025.45140
https://apb.tbzmed.ac.ir

The Kruskal-Wallis test statistics in the Apomorphine-induced climbing behavior test with Low 33a doses (n = 8–10 animals per group), High 33a doses (n = 9–14 animals per group), in the

**Variable**	**H/F-statistic**	**p-value**
**Apomorphine-induced climbing behavior**		
Low doses 33a	17.53	0.0036
High doses 33a	28.35	<0.0001
**Apomorphine-induced yawning**		
Apomorphine dose 0.1 mg/kg	8.421	0.1345, NS
Apomorphine dose 0.032 mg/kg	39.45	<0.0001
**5-HTP-induced head twitches**		
Total head twitches	4.34	0.0049
Dynamic of twitching		
Time factor	5.48	0.0058
Group factor	4.34	0.0049

Apomorphine-induced yawning test with apomorphine dose 0.1 mg/kg (n = 9–10 animals per group), with apomorphine dose 0.0032 mg/kg (n = 9–10 animals per group). The one-way ANOVA test statistics of Total twitches in the 5-HTP-induced head twitches test (n = 8–10 animals per group) and the two-way ANOVA test statistics of Dynamic of twitching.
